# Persistent hepatocyte secretory failure in lopinavir/ritonavir related to drug-induced liver injury: a case report

**DOI:** 10.3389/fmed.2025.1492002

**Published:** 2025-02-06

**Authors:** Yue Sun, Ran Wang, Cai'e Wang, Xiaodong Shao, Xiaojie Zheng, Hui Li, Yingkai Chi, Baocheng Deng, Yiling Li, Shenghao Jin, Xingshun Qi

**Affiliations:** ^1^Department of Gastroenterology, General Hospital of Northern Theater Command (Teaching Hospital of Dalian Medical University), Shenyang, China; ^2^Department of Gastroenterology, General Hospital of Northern Theater Command, Shenyang, China; ^3^Department of Gastroenterology, General Hospital of Northern Theater Command (Teaching Hospital of Shenyang Pharmaceutical University), Shenyang, China; ^4^Department of Gastroenterology, The Second Affiliated Hospital of Shenyang Medical College, Shenyang, China; ^5^Department of Pathology, General Hospital of Northern Theater Command, Shenyang, China; ^6^Department of Infectious Diseases, The First Affiliated Hospital of China Medical University, Shenyang, Liaoning, China; ^7^Department of Gastroenterology, The First Affiliated Hospital of China Medical University, Shenyang, Liaoning, China; ^8^School of Clinical Medicine, China Medical University, Shenyang, Liaoning, China

**Keywords:** case report, drug-induced liver injury, persistent hepatocyte secretion failure, lopinavir/ritonavir, updated RUCAM

## Abstract

Lopinavir/ritonavir, an anti-severe acute respiratory syndrome coronavirus 2 (SARS-CoV-2) drug, may be associated with the development of liver injury. In this paper, we reported an elderly female patient with drug-induced liver injury secondary to lopinavir/ritonavir, which was evaluated for their causality using the updated Roussel Uclaf Causality Assessment Method (RUCAM) of 2016. She had a RUCAM score of 8 which was equivalent to a probable causality grading. Her clinical course was complicated by persistent hepatocyte secretion failure (PHSF), followed by septic shock and SARS-CoV-2 re-infection during her hospitalization. Her response to any medical intervention, including ursodeoxycholic acid, glutathione, methylprednisolone sodium succinate, rifampicin, artificial liver support, and endoscopic nasobiliary drainage (ENBD) was very poor, and her family members refused liver transplantation. Finally, she died. In summary, this case suggests the possibility that lopinavir/ritonavir can cause DILI and even PHSF in our clinical practice.

## Introduction

Drug-induced liver injury (DILI) refers to liver damage caused by drugs and/or their metabolites, which is associated with hypersensitivity or decreased tolerance to drugs during medications. Its main clinical manifestations include abnormal liver tests (LTs) and jaundice, while a few patients may also experience fever, rash, and eosinophilia (1). Its diagnosis is based on a history of hepatotoxic drug use in combination with imaging data after excluding other causes of abnormal LTs (2). Abnormal LTs are necessary for the diagnosis of DILI, but unspecific, including one of the following liver function parameters with their respective thresholds: 1) alanine aminotransaminase (ALT) ≥5 times the upper limit of normal (ULN); or 2) alkaline phosphatase (ALP) ≥2 times ULN, especially in the case of elevated γ-glutamyl transpeptidase (GGT) after exclusion of bone diseases that also may cause elevated ALP (3, 4). Currently, the original Roussel Uclaf Causality Assessment Method (RUCAM) published in 1993 and the updated RUCAM in 2016 are the most widely used tools for diagnosing DILI (5, 6). Early diagnosis and withdrawal of suspected drugs are very important for the treatment of DILI (1).

Lopinavir/ritonavir, a combination drug used to treat severe acute respiratory syndrome coronavirus 2 (SARS-COV2), may be a risk factor for severe liver damage (7). In a study including 148 patients with coronavirus disease 2019 (COVID-19), the use of lopinavir/ritonavir was highly related to liver injury (8). In another cohort study of 1040 COVID-19 patients, the use of lopinavir/ritonavir was associated with an increase in the ratio of ALT to aspartate aminotransaminase (AST), which is an important cause of abnormal liver function and indicates worse outcome (9). In a systematic review, 393 of 996 cases developing liver injury caused by drugs for COVID-19 were verified as the existence of DILI by RUCAM (10). Meanwhile, it also indicated the possibility of DILI caused by lopinavir/ritonavir in COVID-19 patients (10).

Persistent hepatocyte secretion failure (PHSF), first proposed by van Dijk et al. in 2015, refers to severe dysfunction of hepatocellular secretion caused by multiple factors, such as drugs, infections, and transient biliary obstruction, resulting in massive cholestasis (11). Even if the pathogenic factors are removed, severe jaundice caused by massive cholestasis may last for several months. The pathogenesis of PHSF remains unclear, but it is probably due to the inhibition of bile synthesis and transport in hepatocytes after acute injury (12).

Herein, we reported a female patient who suffered fatal DILI secondary to lopinavir/ritonavir, then developed PHSF with many other serious complications during her hospitalization, and eventually died.

## Case presentation

A 72-year-old female patient was admitted to her local hospital due to progressive jaundice on June 26, 2023. In early June 2023, she was diagnosed with COVID-19 and treated with lopinavir/ritonavir. On June 26, laboratory tests showed that total bilirubin (TBIL) was 76.2 μmol/L (reference range: 0–21.0 μmol/L), ALT was 404 U/L (reference range: 0–50 U/L), AST was 277 U/L (reference range: 0–40 U/L), and GGT was 1,290 U/L (reference range: 7–45 U/L). After symptomatic treatment, jaundice was not relieved and TBIL level persistently increased. On July 13, 17, 21, and 25, she was repeatedly treated with dual plasma molecular adsorption system. Since July 24, she was given intravenous infusion of methylprednisolone sodium succinate at a dosage of 40 mg/d, but TBIL level was not improved. Laboratory tests on July 26 showed that TBIL was 444.3 μmol/L, ALT was 324 U/L, AST was 217 U/L, ALP was 712 U/L (reference range: 50–135 U/L), and GGT was 678 U/L. Meanwhile, she presented with somnolence, weakness, and poor appetite.

On July 31, 2023, the patient was transferred to our department. Notably, prior to our admission, she had stopped taking lopinavir/ritonavir. Laboratory tests showed that TBIL was 614.8 μmol/L, direct bilirubin (DBIL) was 393.4 μmol/L (reference range: 0–8.0 μmol/L), ALT was 454.30 U/L, AST was 178.62 U/L, ALP was 955.75 U/L, GGT was 831.63 U/L, albumin (ALB) was 31.0 g/L (reference range: 40–55 g/L), prothrombin time (PT) was 11.7 s (reference range: 9.0–13.0 s), and international normalized ratio (INR) was 1.02 (reference range: 0.8–1.2). Autoimmune liver diseases related antibodies were re-checked, but all of them were negative. She had a 30-year history of hypertension and took nifedipine sustained-release tablet, but denied any other medical or drug use history. Her updated RUCAM score was 8, suggesting a probable causality grading of DILI for lopinavir/ritonavir. Intravenous infusion of methylprednisolone sodium succinate 40 mg/d, glutathione 1.8 g/d, and polyene phosphatidylcholine 465 mg/d was given in combination with oral administration of ursodeoxycholic acid (UDCA) capsule 0.75 g/d. However, severe cholestasis still persisted. The possibility of hereditary liver diseases was also screened. Heterogeneous ABCC6 gene mutation was found. Besides, given her clinical manifestations and disease history, a diagnosis of PHSF was also considered. Thus, she was given 0.3 g/d rifampicin on August 2, 2023. To further rule out the probability of vanishing bile duct syndrome, she also underwent CT-guided percutaneous liver biopsy. Liver histology showed cholestasis of hepatocyte bile capillaries, but bile duct was visible without evidence of bile duct obstruction or chronic liver injury.

On August 4, the patient developed fever with the highest body temperature of 38°C, and then was treated with antipyretics. At the same day, laboratory tests showed that TBIL was 597.8 μmol/L, DBIL was 391.9 μmol/L, ALT was 438.04 U/L, AST was 262.07 U/L, ALP was 1,011.90 U/L, GGT was 732.96 U/L, ALB was 32.0 g/L, PT was 11.4 s, and INR was 0.99. Generally, she had a poor response to drugs for improving cholestasis. Thus, endoscopic retrograde cholangiography (ERC) with endoscopic nasobiliary drainage (ENBD) was performed on August 10, 2023. After that, yellow clarified bile was drained out. On August 11, laboratory tests showed that TBIL was 555.3 μmol/L, DBIL was 409.5 μmol/L, ALT was 107.29 U/L, AST was 93.63 U/L, ALP was 696.42 U/L, GGT was 603.43 U/L, ALB was 31.0 g/L, PT was 11.6 s, and INR was 1.01.

On August 13, she still presented with jaundice, and thus plasma exchange was re-considered. However, at the same day, she developed fever again with a body temperature of 38.9°C, but without chills. SARS-CoV-2 nucleic acid test was positive without other respiratory virus infection. Her family members refused azvudine or other antiviral drugs for COVID-19, because they were considered as a risk of hepatotoxicity and might further worsen liver damage.

**Figure 1:**
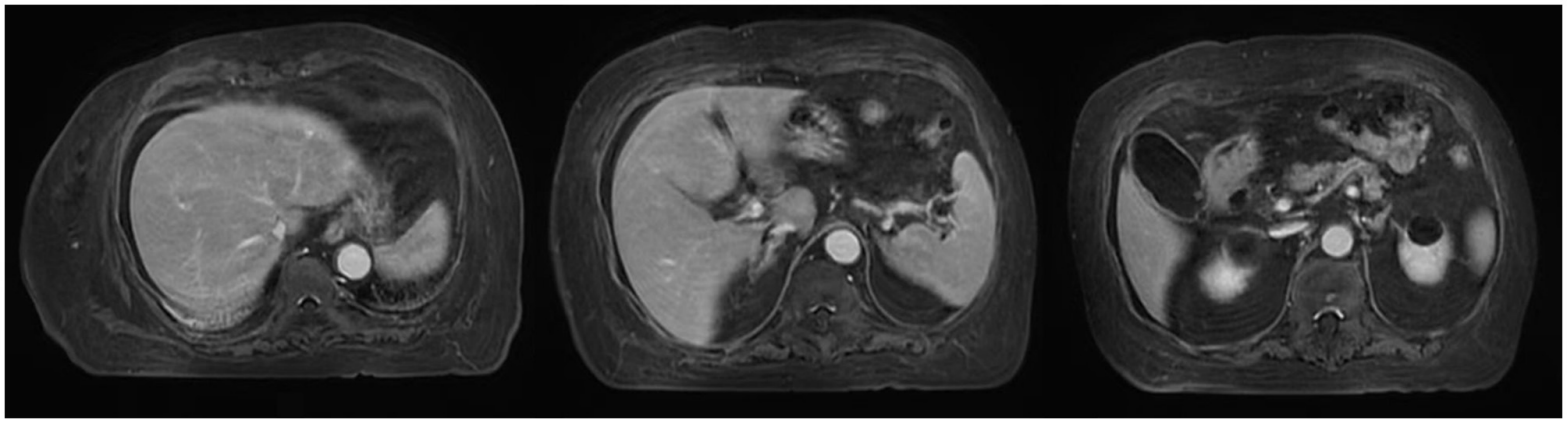
MRI scans in this patient.

**Figure 2:**
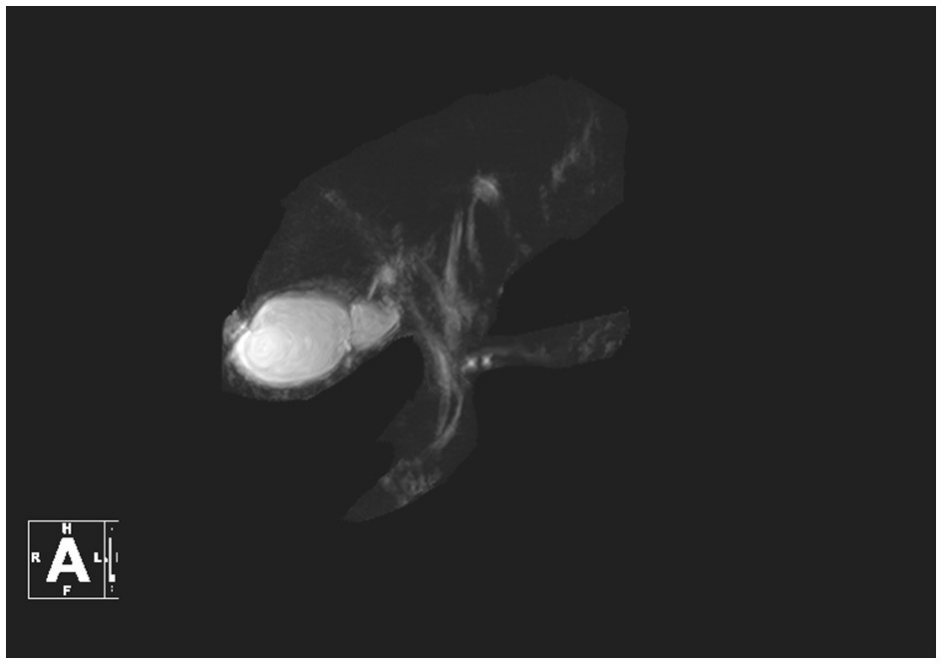
MRCP in this patient.

On August 14, contrast-enhanced MRI (Figure 1) and MRCP (Figure 2) examinations were performed, indicating mild abdominal fluid without any obvious hepatic and bile duct abnormality.

Since August 15, the patient developed fever and chills with a pulse rate of 126/min, a blood pressure of 60/40 mmHg, the highest body temperature of 40.1°C, an elevated procalcitonin level of 10.20 ng/ml (reference range: < 0.05 ng/ml), and a significantly decreased white blood cell count of 1.2 × 10^9^/L containing a lymphocyte count of 0.67 × 10^9^/L and a neutrophil count of 0.3 × 10^9^/L, suggesting the possibility of septic shock. Thus, meropenem and vancomycin were subsequently given. Since then, her body temperature gradually normalized. Blood culture results obtained on August 22 suggested carbapenem-resistant Escherichia coli infection. Thus, amikacin sulfate combined with tigecycline was intravenously given.

**Figure 3:**
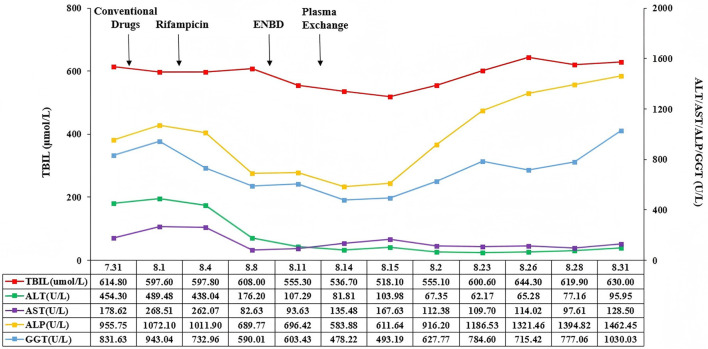
Changes of TBIL, AST, ALT, ALP, and GGT in this patient. TBIL, total bilirubin; AST, aspartate aminotransferase; ALT, alanine aminotransferase; ALP, alkaline phosphatase, GGT, γ-glutamyl transpeptidase.

On August 23, 2023, there was no bile outflow from the nasobiliary drainage. Laboratory tests showed that TBIL was 600.6 μmol/L, DBIL was 474.6 μmol/L, ALT was 62.17 U/L, AST was 109.70 U/L, ALP was 1,186.53 U/L, GGT was 784.60 U/L, ALB was 33.1 g/L, PT was 12.6 s, and INR was 1.10. The patient's general conditions persistently deteriorated (Figure 3). On September 4, 2023, her family members refused further treatment and liver transplantation. After that, she developed multiple gastrointestinal bleeding events. On September 22, 2023, she died at home.

## Discussion

A positive SARS-CoV-2 nucleic acid test is the gold standard for diagnosing COVID-19 (13). Lopinavir/ritonavir, a combined inhibitor of human immunodeficiency virus protease, has been used for the management of acquired immune deficiency syndrome (14, 15). During the COVID-19 epidemic, it has also become a candidate drug for the treatment of COVID-19 (7).

However, its hepatotoxicity should be noted. As we know, drug metabolism includes phase I and phase II reaction. Phase I reaction introduces polar groups through oxidation, reduction, and other enzymatic reactions mainly *via* cytochrome P450 (CYP450) to improve the water solubility of drugs; and phase II reaction further increases the polarity of the drugs and promotes excretion through the binding reaction (16). Subsequently, the post-metabolized product or the unmetabolized drug is actively excreted from the cell *via* the adenosine triphosphate (ATP)-binding cassette (ABC) transporter (17). There are a variety of CYP450 isoenzymes in the liver, among which CYP3A4 accounts for 30% of the total CYP enzymes in the liver (17). Lopinavir/ritonavir is an inhibitor of CYP3A4 and CYP2D6, which affects the metabolism of drugs and leads to liver damage (18, 19). Additionally, lopinavir/ritonavir also inhibits many ABC transporters, including ABCC2, ABCB1, ABCB3, and ABCB11 (20–22). Collectively, liver injury caused by lopinavir/ritonavir develops. Cai et al. (23) found that lopinavir/ritonavir could lead to a four-fold increase in the risk of liver injury among the COVID-19 patients. But liver damage can be rapidly improved after the discontinuation of lopinavir/ritonavir (24). Therefore, lopinavir/ritonavir is potentially hepatotoxic, and should be cautiously used in clinical practice to avoid the development of DILI (25). In our case, DILI caused by lopinavir/ritonavir is considered, based on the interval from the start of this drug to the onset of illness, changes of biochemical indexes, and exclusion of other causes of liver injury (6).

Classification of DILI can guide the choice of treatment. According to the level of liver enzymes, DILI can be divided into three following types: 1) hepatocellular type, characterized as ALT ≥5 times ULN or a ratio of ALT to ALP ≥ 5; 2) cholestatic type, characterized as ALP ≥2 times ULN or a ratio of ALT to ALP ≤ 2; and 3) mixed type, characterized as a ratio of ALT to ALP ranging from 2 to 5 (3). The most important treatment for DILI is to stop using hepatotoxic drugs. Besides, liver-protective drugs, such as N-acetylcysteine, glycyrrhizic acid preparation, bicyclol, polyene phosphatidylcholine, and silymarin, are usually effective for hepatocyte type DILI; anti-cholestasis drugs, such as UDCA and S-adenosylmethionine, are usually effective for cholestasis type DILI; and two or more liver-protecting drugs for mixed type (26). In addition, glucocorticoids are also effective for the treatment of DILI, but they have many adverse effects and should be cautiously prescribed (1, 26). When DILI progresses, such as acute liver failure and hepatic encephalopathy, liver transplantation should be considered (1). Our case was diagnosed with cholestasis type DILI, but poorly responded to UDCA, glucocorticoids, and artificial liver therapy.

The diagnostic criteria for PHSF include: 1) TBIL level > 255 μmol/L; 2) a persistent increase of TBIL level for more than one week after eliminating the potential inducing factors; 3) exclusion of obstructive cholestasis by imaging; and 4) no previous history of chronic liver disease (11). Besides, genetic mutations may be related to the development of PHSF. ABC transporters are a membrane protein superfamily composed of 48 members, which can transport a variety of biological substances through lipid membranes (27). Dysfunction of ABC transporters may be associated with impaired bile formation or excretion, leading to hyperbilirubinemia and cholestasis (27). Bile salt export pump (BSEP) encoded by ABCB11 gene is critical for mediating the transportation of bile acid into the bile duct (28). Multidrug resistance protein 3 (MDR3) encoded by ABCB4 gene promotes the transport of phosphatidylcholine into bile, and phospholipid flippase encoded by ATP8B1 gene is essential for the MDR3 function (28). What's more, uridine diphosphate glucuronosyl transferase 1A1 (UGT1A1) encoded by UGT1A1 gene is mainly responsible for glucosylation of bilirubin (29). UGT1A1 gene mutation can lead to the reduction or complete disappearance of UGT1A1 activity, eventually resulting in hyperbilirubinemia (29). Collectively, hepatocyte and bile duct epithelium can be prevented from bile acid toxicity through BSEP-mediated bile acid excretion and MDR3-mediated bile transportation, while UGT1A1 contributes to the normalization of bilirubin level (28, 29). Defective gene variants or mutations in the canalicular transporter genes ATP8B1, ABCB11, and/or UGT1A1 potentially serve as genetic risk factors of PHSF (11, 12) as well as ABCB4 deficiency (30). Despite our case was diagnosed with PHSF according to the above diagnostic criteria, her genetic test did not demonstrate ATP8B1, ABCB11, UGT1A1, or ABCB4 mutation, but heterogeneous ABCC6 gene mutation, suggesting that ABCC6 gene mutation was probably associated with PHSF. It has been shown that the ABCC6 gene is responsible for maintaining serum inorganic pyrophosphate (PPi) homeostasis, which is a major inhibitor of ectopic calcification (31). Thus, ABCC6 gene mutation leads to the deficiency of PPi and the development of ectopic calcification (31), and it is mainly associated with pseudoxanthoma elasticum (PXE), a rare genetic disease characterized by atherosclerosis and ectopic calcification of connective tissue (31). ABCC6 gene plays an important role in maintaining cholesterol homeostasis, and cholesterol is one of the important components of bile. Therefore, the disorder of cholesterol metabolism caused by ABCC6 gene mutation may indirectly affect bile metabolism, which may be another cause of PHSF (32). In addition, it has been shown that ABCC6 gene affects bile acid levels by affecting the expression of ABCG5 and ABCG8 in mice models (31). However, the current knowledge is very limited regarding the contribution of ABCC6 gene mutation to abnormal bile metabolism and development of PHSF in humans. In future, more comprehensive investigations are warranted to enhance the diagnostic performance of genetic mutation testing in patients with PHSF.

Rifampin, a typical human pregnane X receptor (PXR) agonist, can induce the expression of Cytochrome P450 3A4 (CYP3A4) by activating PXR, and then transform CYP3A4 into a more hydrophilic conjugate catalyzed by enzymes, such as UGT1A1, thereby enhancing the binding and excretion of bilirubin (33). PXR activates the expression of multidrug resistance associated protein 2 (MRP2) encoded by ABCC2, which is responsible for the transportation of bilirubin glucuronide into bile for metabolism (33). PXR activation also upregulates transporters, such as P-glycoprotein, which ultimately transport detoxified bile acid metabolites into bile or urine (33). Thus, rifampicin may be an effective choice of treatment for PHSF (11, 12). van Dijk et al. (11) found that TBIL levels were significantly reduced among 13 patients with PHSF after rifampicin treatment. Similarly, Shi et al. also showed that TBIL levels normalized in 14 of 16 patients with PHSF after rifampicin treatment (12). Unfortunately, rifampicin was ineffective in our case. Based on the current findings, it is only effective for cholestasis caused by the UGT1A1, ABCB11, and ABCC2 gene mutations (12). By comparison, in our case, only ABCC6 gene mutation was detected, and there were multiple serious complications during her hospitalization, which may be a major cause for poor response to rifampicin.

ENBD might be considered in patients with PHSF who had a poor response to rifampicin (12). Shi et al. (12) reported that persistent cholestasis disappeared and TBIL levels normalized after nasobiliary drainage in two patients with PHSF who didn't respond well to rifampicin. However, ENBD was of limited efficacy in our case.

If liver transplantation is not performed, PHSF may threaten the patient's life. However, until now, no studies have explored the efficacy of liver transplantation for PHSF. Regardless, the patient's family refused liver transplantation.

## Conclusion

It should be recognized that lopinavir/ritonavir can cause DILI, and should be immediately discontinued in the case where a diagnosis of DILI is suspected. Moreover, PHSF should be considered, if severe jaundice still persists after stopping hepatotoxic drugs and the response to conventional anticholestatic drugs and glucocorticoids is poor. In future, it should be warranted to explore the efficacy and safety of rifampicin, ENBD, and liver transplantation treatment on PHSF.

## Data Availability

The original contributions presented in the study are included in the article/supplementary material, further inquiries can be directed to the corresponding author.
